# Sepsis in AIDS patients: clinical, etiological and inflammatory characteristics

**DOI:** 10.7448/IAS.16.1.17344

**Published:** 2013-01-30

**Authors:** João Manoel Silva, Sigrid De Sousa dos Santos

**Affiliations:** 1 Department of Infectious and Parasitic Diseases, Faculdade de Medicina da Universidade de São Paulo, São Paulo, Brazil; 2 Department of Medicine, Universidade Federal de São Carlos, São Carlos, Brazil

**Keywords:** sepsis, HIV, AIDS, etiology, inflammatory markers, mortality

## Abstract

**Introduction:**

Intensive care mortality of HIV-positive patients has progressively decreased. However, critically ill HIV-positive patients with sepsis present a worse prognosis. To better understand this condition, we propose a study comparing clinical, etiological and inflammatory data, and the hospital course of HIV-positive and HIV-negative patients with severe sepsis or septic shock.

**Methods:**

A prospective observational study enrolling patients with severe sepsis or septic shock associated or not with HIV infection, and admitted to intensive care unit (ICU). Clinical, microbiological and inflammatory parameters were assessed, including C-reactive protein (CRP), procalcitonin (PCT), interleukin-6, interleukin-10 and TNF-α. Outcome measures were in-hospital and six-month mortality.

**Results:**

The study included 58 patients with severe sepsis/septic shock admitted to ICU, 36 HIV-positive and 22 HIV-negative. All HIV-positive patients met the criteria for AIDS (CDC/2008). The main foci of infection in HIV-positive patients were pulmonary and abdominal (*p*=0.001). Fungi and mycobacteria were identified in 44.4% and 16.7% of HIV-positive patients, respectively. In contrast, the main etiologies for sepsis in HIV-negative patients were Gram-negative bacilli (36.4%) and Gram-positive cocci (36.4%) (*p*=0.001). CRP and PCT admission concentrations were lower in HIV-positive patients (130 vs. 168 mg/dL *p*=0.005, and 1.19 vs. 4.06 ng/mL *p*=0.04, respectively), with a progressive decrease in surviving patients. Initial IL-10 concentrations were higher in HIV-positive patients (4.4 pg/mL vs. 1.0 pg/mL, *p*=0.005), with moderate accuracy for predicting death (area under receiver-operating characteristic curve =0.74). In-hospital and six-month mortality were higher in HIV-positive patients (55.6 vs. 27.3% *p*=0.03, and 58.3 vs. 27.3% *p*=0.02, respectively).

**Conclusions:**

The course of sepsis was more severe in HIV-positive patients, with distinct clinical, etiological and inflammatory characteristics.

## Introduction

Antiretroviral therapy has improved life expectancy and reduced morbidity of HIV-positive patients [1]. However, the decline in AIDS-related diseases has increased the importance of illnesses not directly related to HIV infection [2, 3]. Among them, sepsis has been responsible for 12-31% of HIV-positive patients’ admissions to intensive care units (ICUs), and is associated with a worse prognosis [4–6].

While the sepsis incidence rate is estimated to be 150–300 cases per 100,000 inhabitants, among patients with chronic diseases, the rate increases to 700 cases per 100,000 patients [7]. In patients with HIV infection, the rate reaches 1,000 cases per 100,000 patients [8]. In spite of this, HIV-positive patients with severe sepsis are less often admitted to ICU, even compared to patients with a similar clinical condition or higher expected lethality [9].

Clinical manifestations of sepsis in immunosuppressed patients can be minimal or non-specific, and the systemic host response to infection is expected to be blunted. In addition, the wider spectrum of infectious agents could require a broader spectrum of antimicrobial regimen. Expert management of sepsis in HIV-positive patients is needed to predict and establish the correct diagnosis and to choose appropriate empiric and specific antimicrobial agents and improve prognosis [10].

Levels of inflammatory biomarkers, C-reactive protein (CRP) and procalcitonin (PCT), have been associated with systemic infection in patients with several immune dysfunctions [11, 12]. However, this response is observed mainly in bacterial sepsis and seems to be less expressive in viral, fungal and mycobacterial infections [13–15], microorganisms frequently found in AIDS patients. Other markers like serum interleukin-6 (IL-6) and interleukin-10 (IL-10) levels have been associated with more severe infection, multiple organ dysfunction and lethal outcome [16–18]. The IL-6/IL-10 ratio seems to indicate a balance in sepsis between pro- and anti-inflammatory cytokines [19]. Interestingly, IL-10 concentration increases with HIV immune dysfunction [20].

The poor innate immune response in HIV-positive patients predisposes them to sepsis [21, 22], with particularities in terms of clinical signs, etiologic agents, inflammatory patterns and prognosis [5, 6, 23]. Therefore, this study aimed at comparing the characteristics and outcomes of HIV-positive and HIV-negative adult patients with severe sepsis or septic shock in a critical care setting.

## Methods

### Study population

A prospective observational study was conducted in a six-bed infectious diseases ICU. Adults with severe sepsis/septic shock, with or without HIV infection, admitted from July 3rd, 2006, through December 3rd, 2008, were enrolled.

The sample size calculation was designed to discriminate the accuracy of biomarkers in predicting death in septic HIV-positive patients. Non-surviving septic patients usually have admission PCT and CRP concentrations significantly higher than survivors, with accuracies determined by the area under their receiver-operating characteristic (ROC) curves of 0.878 and 0.811, respectively [24]. However, this response has varied in different studies according to host, clinical situation, etiologic agent and source of infection [25]. By defining the “high capability” of a biomarker in predicting death in septic HIV-positive patients as an area under its ROC curve (AUC) of at least 0.8, with a null hypothesis value of 0.5, a type I error-alpha of 5% and a power of 80%, a sample size of at least 30 septic HIV-positive patients was predicted to be necessary, using an expected mortality of 50% [5, 26]. An HIV-negative control group was created for comparison purposes with an HIV-positive/negative ratio of 2:1.

Eligible subjects were≥18 years of age, with severe sepsis or septic shock (1992 consensus) [27]. We excluded patients with life expectancy less than 24 hours, more than 24 hours of antibiotic therapy, previous liver or renal failure, in the immediate postoperative period or with another cause of immunosuppression; all factors that could interfere with the biomarkers’ prognostic value, metabolism [28, 29], basal level [30] and response to injury [31].

The study protocol was approved by the Investigational Review Board of Hospital das Clínicas, and written consent was obtained from all participants. The clinical trial was also registered in the National System of Information about Ethics in Research – SISNEP (CAAE - 3011.0.015.000-05).

## Data collection

After admission to the study, all patients with unknown HIV serological status were submitted to HIV serological testing (ELISA and Western Blot), and patients were then classified into two groups according to HIV serodiagnosis.

A data entry form was created with standardized information on sex, age, infection severity, number and time to onset of organic dysfunctions, Acute Physiology and Chronic Health Evaluation Score II (APACHE II) [32, 33], primary focus and infection agent [34], need for respiratory or hemodynamic invasive support in the first 24 hours of admission, length of hospital stay before ICU admission and laboratory findings.

Infection severity was graded as severe sepsis or septic shock based on the reversibility of organ dysfunction with fluid resuscitation (1992 consensus) [27]. Agents were identified and classified as Gram-negative bacteria, Gram-positive bacteria, fungi, parasites, and viruses. The criteria used for the diagnosis of infectious foci and agents were made and revised according to CDC guidelines [34, 35].

HIV-positive patients were also evaluated for HIV risk factors, duration of HIV infection, previous or current diagnosis of AIDS-defining illnesses, CD4 lymphocyte count, plasma HIV-1 RNA load, and use of antiretroviral medications.

All patients were then submitted to routine blood sample collection, including determinations of C-reactive protein, procalcitonin, interleukin-6, interleukin-10 and tumour necrosis factor-α (TNF-α). HIV-positive patients were also submitted to blood sampling for plasma RNA (HIV-1) quantification and immunophenotyping of blood lymphocytes at admission.

Cytokines present early peaks and short half-lives after septic stimulus [36]. Blood sampling for cytokine assays was conducted at admission entry (D1) and on the seventh (D7) day. The biomarkers CRP and PCT, in contrast, continue to increase for two or three days after an infectious stimulus [37]. For this reason, sampling for C-reactive protein and procalcitonin assays was conducted at admission entry (D1) as well as on Day 3 (D3) and Day 7 (D7). The Day 7 sample was included to better evaluate the response to treatment.

All patients were followed until death or hospital discharge. Participants were contacted personally or by telephone to complete six months of follow-up.

## Laboratory analysis

Serum PCR determination was performed by routine nephelometry technique. Plasma PCT determination was performed by Manual Chemiluminescence PCT Assay (LUMItest^®^ PCT, BRAHMS AG, Hennigsdorf). Determinations of interleukin-6, interleukin-10, and TNF-α cytokine concentrations were performed by enzyme immunoassay (Biotrak Easy ELISA).

## Statistical analysis

Data were collected in a Microsoft^®^ Excel database and analyzed through Statistical Package for Social Sciences 13.0 (SPSS 13.0) and MedCalc 11.5.1 [26, 38]. Demographic, clinical, microbiological, immunological and inflammatory entry parameters were compared between groups. Continuous variables that showed skewed distribution were assessed by Mann–Whitney test for comparing two variables, and by Friedman test for more than two variables. Student *t*-test was used for continuous variables with normal distribution. Categorical variables were analyzed by Chi-square test and Fisher's exact test. Friedman repeated measures test was applied to assess trends in the biomarkers evolution from admission to the seventh day in surviving patients. All statistical tests were two-tailed, with a significance level of 0.05.

Cox proportional hazard models were performed to identify predictors of 28-day and six-month mortality. Variables were included in the model, if their significance level was p<0.20 or were clinically relevant. A p-value of ≤0.05 was considered statistically significant.

The accuracy of the different biomarkers in predicting death was compared through ROC analysis. Biomarkers were then classified as either non-informative (AUC=0.5), less accurate (0.5<AUC≤0.7), moderately accurate (0.7<AUC≤0.9), highly accurate (0.9<AUC<1) or perfect tests (AUC=1) [39].

## Results

### Demographic and clinical characteristics

A total of 113 septic patients, 54 HIV-positive and 59 HIV-negative, were admitted to ICU from July 3rd, 2006, through December 3rd, 2008. However, 55 patients, 18 HIV positive and 37 HIV negative, were excluded for different reasons (12 for previous use of antibiotics, 11 for other causes of immunosuppression, nine end-stage renal failures, seven for immediate postoperative periods, eight severely ill patients with anticipated life expectancy less than 24 hours, seven refutations of informed consent and one for liver failure).

Among the 58 patients enrolled, 36 were HIV-positive and 22 were HIV-negative (Table 1). Despite there being no significant difference between the groups with respect to sex and age, HIV-positive patients tended to be older and male. All HIV-positive patients presented with manifest disease (AIDS), 58.7% of them with an AIDS-defining illness at the time of ICU admission, median CD4 count of 25 cells/mm^3^ (12.5–100.5 cells/mm^3^), and median log RNA (HIV-1) of 5.1 (4.6–5.7). HIV infection was newly diagnosed in 57.1% of the patients. Median duration of HIV infection was 83 days (10–360). Only 42.9% of the patients had prior use of antiretroviral therapy. Median duration of antiretroviral therapy was 360 days (56.5–362.5).

**Table 1:** Clinical and laboratory characteristics of HIV-positive and HIV-negative septic patients admitted to study

Characteristic	HIV-positive (*n*=36)	HIV-negative (*n*=22)	P*
Age – mean years±SD	39.5±9.8	46.3±18.0	0.07
Sex, n (%)			0.09
Male	24 (66.7)	10 (45.5)	
Female	12 (33.3)	12 (54.5)	
Sepsis severity			
Severe sepsis (%)	44.4	45.5	0.58
Septic shock (%)	55.6	54.5	0.58
Prognostic scores			
APACHE II	21.5±7.9	22.3±6.2	0.67
SOFA	8.8±4.6	7.8±3.1	0.37
Number of organic dysfunctions	4 (2–5)	3 (2–5)	0.83
Suspected focus of infection (%)			0.001
Pulmonary	83.3	40.9	
Central nervous system	1.8	17.8	
Bloodstream	5.6	13.6	
Urinary	0.0	13.6	
Dermatologic	0.0	12.3	
Abdominal	8.3	0.0	
Etiologic agent (%)			0.001
Bacteria			
Gram– rods	8.3	36.4	
Gram+ cocci	27.8	36.4	
Fungi	44.4	9.1	
Mycobacteria	16.7	0.0	
Unknown	2.8	18.2	
Mechanical ventilation on first day (%)	58.3	63.6	0.52
Vasopressors on first day (%)	61.1	54.5	0.41
Lactate (mmol/L)	2.9±1.3	2.8±1.4	0.84
Glucose (mg/dl)	125.5±75.5	135.2±57.9	0.61
Cortisol (µg/dL) median (25–75 IQR)	17.9 (10.3–55.7)	16.7 (6.3–36.2)	0.95
WBC (cell/mm^3^) median (25–75 IQR)	5,555 (3,010–12,000)	19,630 (13,140–25,950)	0.02
Time from organic dysfunction onset (hours)	15 (8.5–24.0)	18 (8.0–24.0)	0.50
Length of hospital stay before ICU admission (hours)	18.0 (16.0–24.0)	15.0 (4.0–24.0)	0.60

* Wilcoxon–Mann–Whitney test; student *t*-test; Chi-square test; Fisher's exact test.

Sepsis severity and prognostic scores were similar at admission between both groups. Whereas the organic dysfunctions most frequently found in HIV-positive patients were respiratory (77.8%), cardiovascular (75%) and neurological (52.8%), in HIV-negative patients the main dysfunctions were cardiovascular (72.7%), renal (63.6%) and lactic acidosis (29.1%).

Pulmonary and abdominal foci were more frequent in HIV-positive patients. Assessing the microorganism identified during the sepsis episode, there was a predominance of fungal and mycobacterial infection in HIV-positive patients, while Gram-positive cocci and Gram-negative rods were more frequently isolated from HIV-negative patients. The fungi identified in HIV-positive patients were *Pneumocystis jirovecii* (eight patients), *Histoplasma capsulatum* (four patients), *Cryptococcus sp* (two patients), and *Candida albicans* (one patient). Despite *Pneumocystis jirovecii* not being associated with systemic repercussions, no other infectious agent could be concomitantly identified in these patients. The only fungus identified in HIV-negative patients was *Candida albicans* (two patients).

The septic HIV-positive patients presented lower WBC counts at admission. There was no difference between the groups regarding a need for vasoactive drugs and/or invasive mechanical ventilation, or length of hospital stay before ICU admission.

## Inflammatory biomarkers

**Figure 1:**
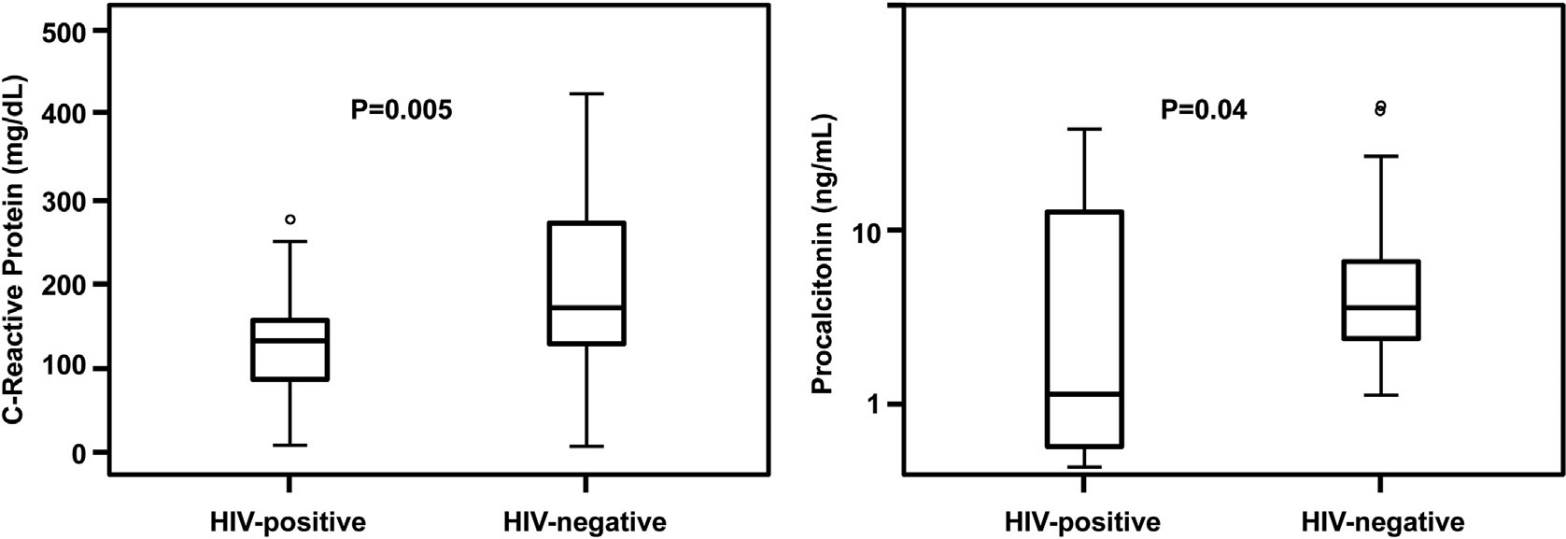
Initial concentrations of C-reactive protein and procalcitonin in HIV-positive and HIV-negative septic patients. Box plot definitions: horizontal bars represent medians; boxes represent interquartile range (IQR); vertical bars represent values between upper and lower outlier limits; upper outlier limit=upper quartile +1.5×IQR; lower outlier limit=lower quartile −1.5×IQR; circles represent outliers. *Wilcoxon–Mann–Whitney test.

**Figure 2:**
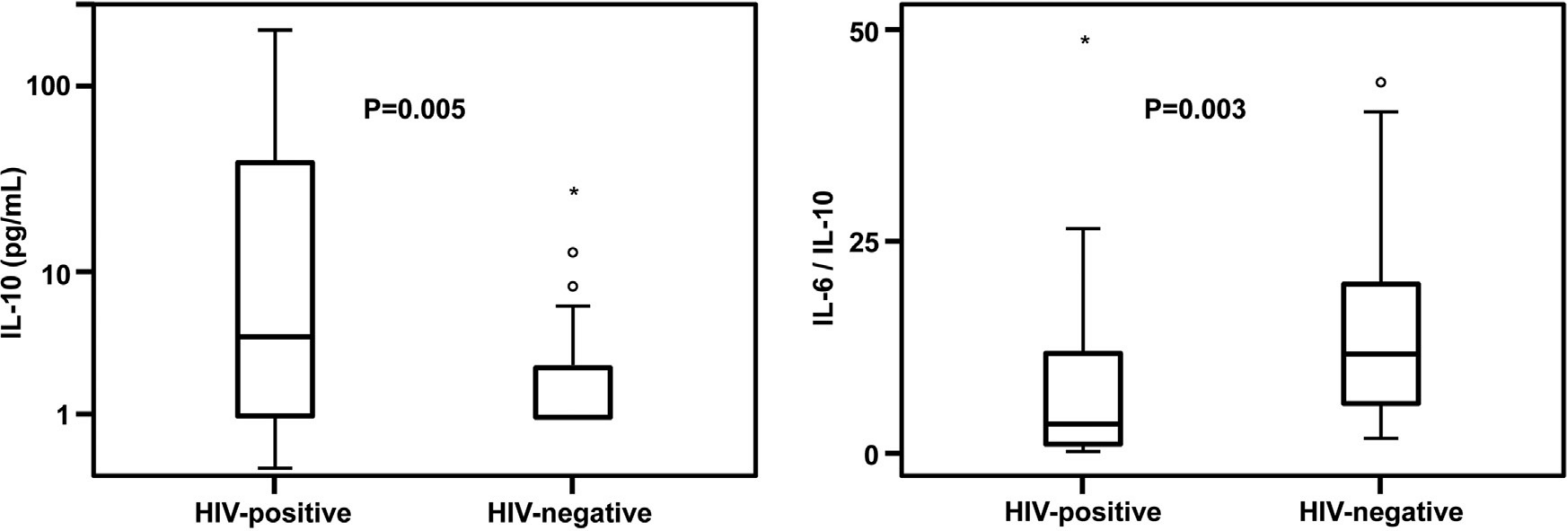
Initial interleukin-10 concentrations and interleukin-6/interleukin-10 ratio in HIV-positive and HIV-negative septic patients. **Box plot definitions: horizontal bars represent medians; boxes represent interquartile range (IQR); vertical bars represent values between upper and lower outlier limit; upper outlier limit=upper quartile+1.5×IQR; lower outlier limit=lower quartile−1.5×IQR; circles represent outliers; plus signs represent extreme outliers. ±Wilcoxon–Mann–Whitney test**.

Initial serum concentrations of CRP and PCT were lower in septic HIV-positive patients (Figure 1). Initial serum concentrations of IL-10 were significantly higher in HIV-positive septic patients than in HIV-negative patients, [4.4 pg/mL (1.0–38.1) vs. 1.0 pg/mL (1.0–2.7), respectively (*p*=0.005)] (Figure 2). Initial IL-6 concentrations were similar between septic HIV-positive and HIV-negative patients [38.0 pg/mL (8.4–45.6) vs. 20.1 pg/mL (9.4–45.0), respectively (*p*=0.52)]. Septic HIV-positive patients presented lower initial IL-6/IL-10 ratios than septic HIV-negative patients [3.5 (0.9–11.7) vs. 11.9 (5.4–20.1), respectively (*p*=0.003)] (Figure 2). Both groups of septic patients maintained TNF-α serum concentrations within normal parameters, that is,<2.5 pg/mL. There was a tendency for a progressive decline in the IL-10 level among HIV-positive septic patients (*p*=0.02) and a tendency for progressive declines in the other biomarkers among HIV-negative septic patients (*p*<0.01) from the first to the seventh day.

## Mortality

Both ICU and hospital mortality were higher in HIV-positive septic patients. Furthermore, after six months of follow-up, there was one additional death among the septic HIV-positive patients (Table 2).

**Table 2:** Clinical outcome of HIV-positive and HIV-negative septic patients

Outcome	HIV-positive (*n*=36)	HIV-negative (*n*=22)	p
ICU length of stay (days)	7.5 (4.5–12.5)	10 (7–15)	0.23
Hospital length of stay (days)	18.5 (10.0–39.5)	22 (14–43)	0.31
ICU mortality (%)	50.0	18.2	0.01
In-hospital mortality (%)	55.6	27.3	0.03
Six-month mortality (%)	58.3	27.3	0.02

Cox regression analysis identified a harmful effect of HIV infection (HR 4.2, 95% CI 1.02–17.10) and of the number of organ dysfunctions (HR 1.38, 95% CI 1.05–1.80) on risk of death within 28 days of follow-up, even after adjusting for potential confounding factors such as sex, age, etiologic agent, WBC counts and organ dysfunction.

Cox regression analysis also verified a sustained detrimental effect of HIV infection (HR 3.73, 95% 1.46–9.53) and of the number of organ dysfunctions (HR 1.54, 95% CI 1.20–1.99) on the risk of death in six months of follow-up, even after adjusting for potential confounding factors, such as sex, age, etiologic agent, WBC counts and organ dysfunction.

The variables sex, age, etiologic agent, leukocyte count and number of organ dysfunctions were introduced in the model because they have presented different distribution patterns in septic patients with or without HIV infection. However, they did not statistically affect 28 days and six-month lethality (Supplementary Tables 3 and Table 4).

**Figure 3:**
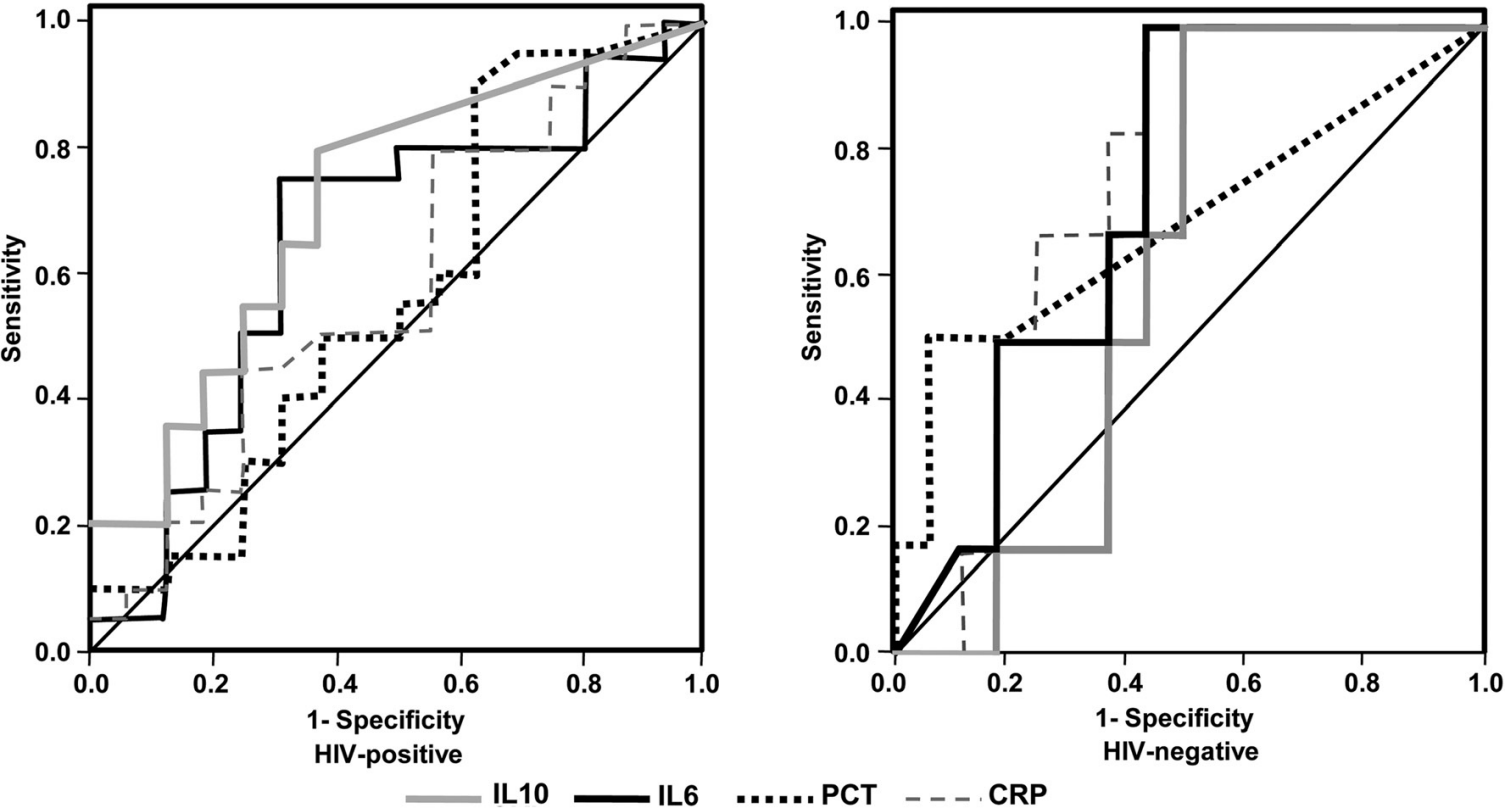
Receiver operating characteristic curves of biomarkers for prediction of hospital mortality in HIV-positive and HIV-negative septic patients. **Definition of abbreviations: CRP, C-reactive protein; PCT, procalcitonin; IL6, interleukin-6; IL10, interleukin-10**.

Most biomarkers showed similar discriminatory power, with low accuracy in predicting the death of septic HIV-positive patients, except for IL-10, which demonstrated a moderate accuracy in predicting in-hospital mortality. The areas under the ROC curves for PCT, CRP, IL-6 and IL-10 on admission were 0.57, 0.58, 0.65 and 0.74, respectively (Figure 3).

In septic HIV-negative patients, all biomarkers except IL-10 presented a moderate discriminatory power for predicting death. The areas under the ROC curves for measurement of IL-10, PCT, IL-6 and CRP on admission were 0.60, 0.70, 0.72 and 0.74, respectively (Figure 3).

**Figure 4:**
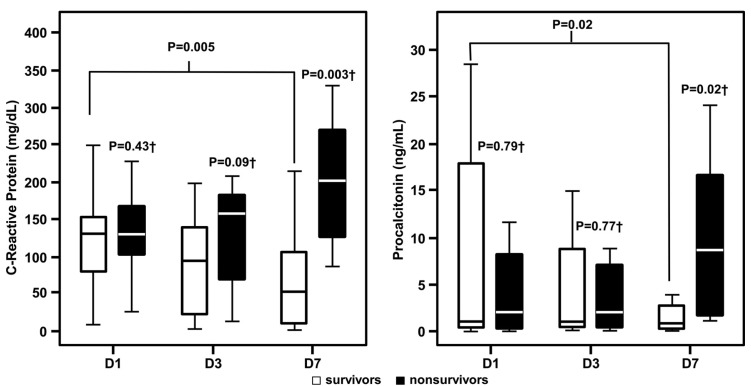
C-reactive protein and procalcitonin concentrations in HIV-positive septic patients according to survival and time of measurement. **D1, admission; D3, third day; D7, seventh day; ±Wilcoxon–Mann–Whitney test comparing survivor and non-survivor patients; † Friedman test for D1, D3 and D7 comparisons between survivor and non-survivor patients**.

Despite the low discriminatory power of admission levels of CRP and PCT in predicting mortality in HIV-positive septic patients, survivors presented progressive declines in these biomarkers on the third and seventh days, compared with increasing levels found in patients who died (Figure 4).

## Discussion

The longer life expectancy of HIV-positive patients due to antiretroviral therapy and the improvement in ICU outcome have stimulated an earlier indication of critical care for this population [40, 41]. Over time, sepsis is becoming more important as a cause of admission of HIV-positive patients to the ICU, and it is associated with a poor outcome [5, 9, 23, 40]. Therefore, it is necessary to understand more fully the particulars of sepsis in this population.

Despite a successful AIDS programme in Brazil, diagnosis of HIV infection is still late, with start of treatment characterized by the presence of a severe immune deficiency or an AIDS-defining disease [42]. Reflecting the late diagnosis of HIV infection, patients admitted to ICU were severely immunocompromised, with manifest AIDS-defining illnesses and low CD4 counts (median 25 cells/mm^3^). However, low CD4 counts may be also associated with the severity of sepsis [43]. Only 42.9% of patients reported previous use of antiretroviral drugs, as previously observed [5]. Moreover, increased ART availability has shown little overall impact on its rate of utilization even in developed countries [4].

Pulmonary and abdominal foci were more associated with sepsis in HIV-positive patients than in HIV-negative patients. The predominance of a pulmonary focus as a cause of sepsis in HIV-positive patients has been observed in other studies [5]. However, the higher frequency of abdominal focus also suggests that further investigation is warranted.

Another remarkable fact was the high proportion of study patients in which an infectious agent could be identified (97.2% in HIV-positive patients and 81.8% in seronegative patients), with higher proportions of fungi and mycobacteria in HIV-positive patients. This was probably due to an aggressive etiological investigation, since these etiologies certainly would not have been evidenced without proper suspicion and diagnostic investigation.

Unusual etiologies and their severe immunosuppressive state may have been responsible for the lower WBC counts observed in HIV-positive septic patients. This could have delayed diagnosis, if other clinical parameters of sepsis had not been closely observed. The in-ICU mortality rate found in this research was similar to previous studies, in which the mortality rate of septic HIV-positive patients in the ICU has varied from 29 to 66% [6, 9, 23].

The mortality of severe sepsis/septic shock was significantly lower in the HIV-negative group (27.3%), composed of younger patients without any known chronic underlying disease or immunosuppression. Despite the mean expected mortality rate of severe sepsis/septic shock usually being higher in the general population, this is not a homogeneous reality and improves with compliance to sepsis guidelines [44, 45]. The severity of infectious episodes is especially higher in patients with chronic underlying disorders [46]. A multicentre retrospective study of 192,980 patients with severe sepsis also found a mortality rate of 28.6%, that increased with age (>100-fold), presence of metastatic neoplasm (43%), non-metastatic neoplasm (37%), chronic liver or renal disease (37%), HIV infection (34%) and chronic obstructive pulmonary disease (32%) [7].

Clinical studies have shown sex disparities in sepsis incidence and outcome. These discrepancies may result from not only study design and biological differences between men and women but also from issues related to gender lifestyle, comorbidities, access to health care services and critical care investment [47]. In this study, despite HIV-positive patients with sepsis tending to be male, sex did not significantly influence mortality.

The inflammatory response shows a different behaviour in HIV-positive septic patients, with lower titres of CRP and PCT, and higher levels of IL-10. Despite both CRP and PCT being known specific markers of infection [48–52], with progressively higher levels in sepsis, severe sepsis and septic shock patients [53, 54], infections caused by fungi, parasites, virus and mycobacteria do not seem to increase the levels of these biomarkers in the way bacterial infection do in HIV-positive patients [55, 56].

IL-10 seems to inhibit not only the production of proinflammatory cytokines and mediators from monocytes/macrophages and dendritic cells, particularly IL-2 and interferon-γ (IFN-γ), but also IL-4 and IL-5 [18]. Higher IL-10 levels are associated with impaired immunity against bacterial, mycobacterial and viral infections. The serum concentration of IL-10 increases in HIV-positive patients with progression to AIDS, and decreases with highly active antiretroviral therapy (HAART) [20]. The higher IL-10 levels observed in HIV-positive patients could explain their poorer response to acute infectious damage, with a worse sepsis outcome.

Initial IL-10 concentrations in HIV-positive septic patients were more accurate for detecting progression to death than levels of CRP, PCT and IL-6. Despite the low accuracy of CRP and PCT in predicting death, persistent high levels of these biomarkers were associated with a poorer prognosis. Therefore, these markers may assist in identifying patients without response to initial antimicrobial treatment, and possibly enable early therapeutic intervention [51, 52].

## Conclusions

Sepsis has a more severe course in HIV-positive patients, with a predominance of pulmonary and abdominal foci, fungal and mycobacterial etiologic agents, and lower responses by inflammatory markers. Consequently, it is important to have a high clinical suspicion for sepsis, with an aggressive etiological investigation and therapeutic response monitoring.
